# Preussianone, a New Flavanone-Chromone Biflavonoid from *Garcinia preussii* Engl

**DOI:** 10.3390/molecules17056114

**Published:** 2012-05-21

**Authors:** Bernadette Biloa Messi, Karine Ndjoko-Ioset, Barbara Hertlein-Amslinger, Alain Meli Lannang, Augustin E. Nkengfack, Jean-Luc Wolfender, Kurt Hostettmann, Gerhard Bringmann

**Affiliations:** 1 Department of Organic Chemistry, University of Yaoundé, P.O. Box 812, Yaoundé, Cameroon; Email: biloa83@yahoo.fr (B.B.M.); ankengf@yahoo.fr (A.E.N.); 2 School of Pharmaceutical Sciences, University of Geneva, Quai Ernest-Ansermet 30, 1211 Geneva 4, Switzerland; Email: jean-luc.wolfender@unige.ch (J.-L.W.); kurt.hostettman@unige.ch (K.H.); 3 Institute of Organic Chemistry, Am Hubland, University of Würzburg, Würzburg 97074, Germany; Email: hertlein@chemie.uni-wuerzburg.de; 4 Department of Chemistry, Higher Teachers’ Training College, University of Maroua, P.O. Box 46, Maroua, Cameroon; Email: alainmeli@yahoo.com

**Keywords:** *Garcinia* biflavonoids, absolute configuration, high-temperature NMR, antibacterial activity

## Abstract

A new flavanone-chromone biflavonoid, preussianone (**1**), has been isolated from the leaves of *Garcinia preussii*, along with four known biflavonoids. The absolute stereostructures were elucidated by chemical, spectroscopic, and chiroptical methods. The biological properties of the new biflavonoid against several bacterial strains were evaluated.

## 1. Introduction

Although the occurrence of biflavonoids is limited to only a few families, they constitute one of the major classes of complex secondary metabolites [1]. These compounds are formed through phenol-oxidative coupling of flavones, flavonols, dihydroflavonols, flavanones, isoflavones, aurones, auronols, or chalcones [1]. Besides homo-coupling, the mixed combination of different phenolic building blocks can lead to a broad variety of chemical structures [1,2].

Biflavonoids are classified into seven major types: agathisflavones, cupressuflavones, amentoflavones, hinoflavones, robustaflavones, miscellaneous synthetic biflavonoids, and *Garcinia* biflavonoids (GBs) [3]. The latter are generated from linkage variations of two monomers, generally leading to C-3/C-8'' biflavanones, C-3/C-8''-linked biflavone, and C-3/C-8''-coupled flavanone-flavone analogs. All these dimers (except for biflavones) carry at least one stereogenic center [4,5,6], but also show atropisomeric behavior due to restricted rotation about the central axis.

Many pharmacological effects of biflavonoids have been assessed, such as their ability to inhibit histamine release [7] and platelet adhesion [8]. Anti-inflammatory [9] as well as antibacterial activity related to biflavones have also been reported [10,11].

**Figure 1:**
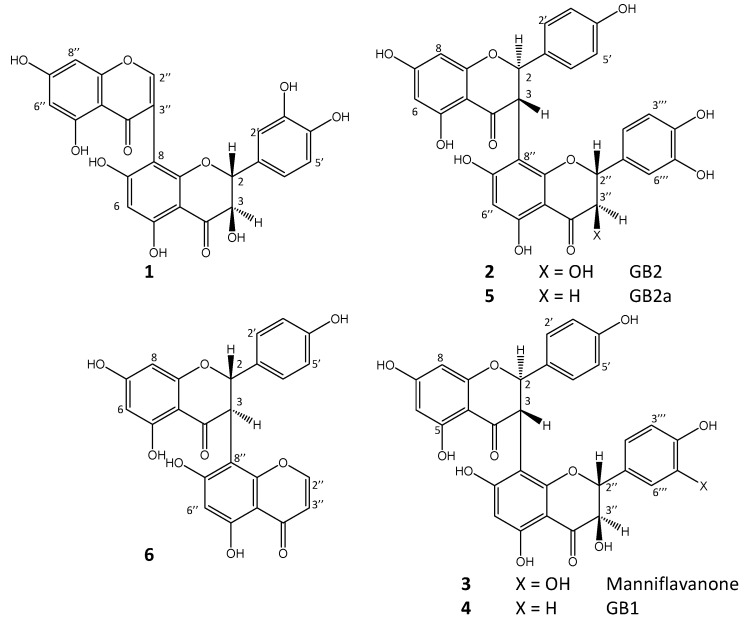
Chemical structures of compounds **1–6**.

The genus *Garcinia* is distributed in tropical regions. It is known to be a rich source of bioactive xanthones, benzophenones, and biflavonoids [12,13,14]. In Africa, *Garcinia preussii* Engl. (*syn* *G. epunctata* Stapf) is traditionally used to treat stomach aches [15]. The leaves are prepared as a decoction to relieve toothache [16]. In the course of a phytochemical survey and biological evaluation of medicinal plants in Cameroon, we undertook the chemical investigation of *G.* *preussii* leaves. This species has not yet been described chemically, nor pharmacologically. In this paper, we report on the isolation of a new mixed flavone-chromone “dimer” named preussianone (**1**), along with some further, known GBs from *G. preussii* [17,18,19]. Their chemical structures (Figure 1) were established by NMR (relative configuration) and circular dichroism (CD) spectroscopy (absolute configuration). In doing so, we clarify some of the structural stereochemical issues published previously about GBs from related plants.

## 2. Results and Discussion

### 2.1. Structure Elucidation

Air dried leaves of *G. preussii* were sequentially extracted with acetone and methanol at room temperature. The crude methanol extract was subjected to column chromatography on silica gel, which led to the isolation of one new chromone-flavonoid **1**, along with four known GBs **2–5** [17,18].

Compound **1** was obtained as yellow oil. The molecular formula was C_24_H_16_O_11_ as deduced from HRESIMS. A maximum observed in the UV spectrum at 290 nm and a shoulder at 340 nm were characteristic of a flavanone derivative.

**Figure 2:**
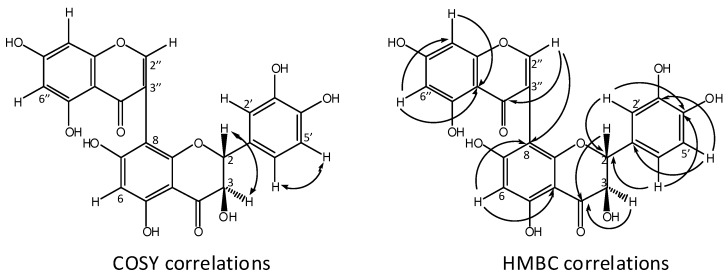
Compound **1**, COSY and HMBC correlations.

Two doublets at *δ*_H_ 4.95 (1H, *d*, 10.7 Hz, H-2) and 4.43 (1H, *d*, 10.7 Hz, H-3) indicated the presence of a dihydroflavonol moiety. By COSY and HMBC correlations (Figure 2), the aromatic protons at *δ*_H_ 6.19 (1H, *d*, 2.1 Hz) and 6.34 (1H, *d*, 2.1 Hz) were assigned to be located at C-6'' and C-8'', respectively. Broad doublets at *δ*_H_ 6.67 (1H, *brs*), 6.67 (1H, *brs*), and 6.80 (1H, *brs*), and the singlet at 6.08 (1H, *s*) were attributed to the positions C-2', C-5', C-6', and C-6, respectively, and confirmed the flavanone moiety. Moreover a singlet at *δ*_H_ 8.13 (1H, *s*, H-2'') and the mass difference related to the assigned flavanone, suggested a chromone building block. The ^13^C-NMR of **1** displayed 11 low-field signals, which corresponded to two carbonyl carbons, six hydroxylated carbons, two aromatic carbons, and one ethylenic carbon bearing an oxygen bridge to the pyran ring of the benzopyran moieties. By a typical [19,20,21] downfield shift of the ^13^C-NMR signal at C-8 (98.2 ppm), along with an upfield shift at C-8'' (93.7 ppm), the axis was assigned to be located at C-3''/C8. The link between the two moieties via C-3'' and C-8 was also confirmed by clear HMBC and COSY interactions (Figure 2).

**Figure 3:**
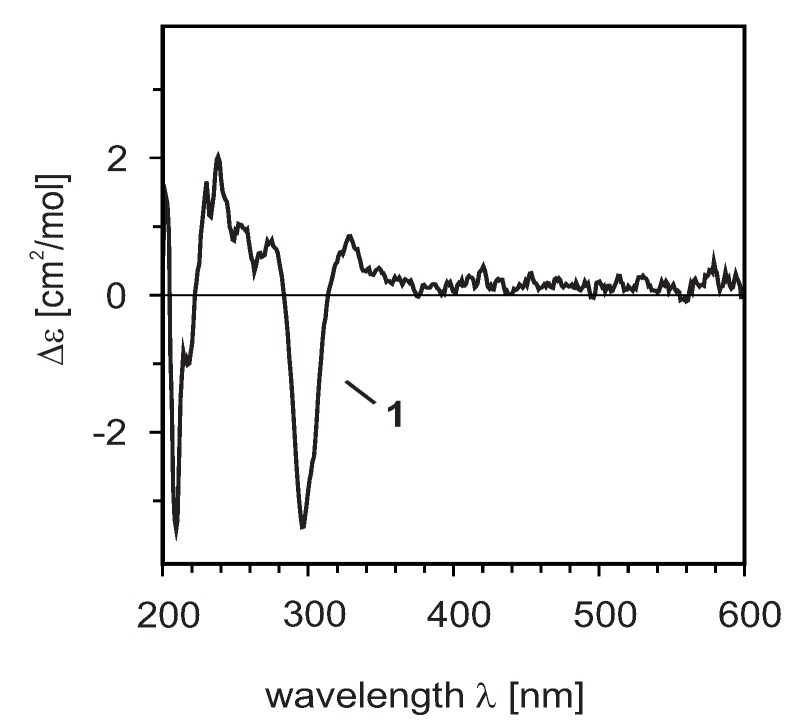
Experimental CD spectrum of compound **1**.

The relative configuration at the two stereogenic centers of compound **1** was deduced to be *trans* from the constant coupling between C-2 (4.95 ppm) and C-3 (4.43 ppm) of 10.7 Hz, showing that H-2 and H-3 were diaxial, so the absolute configuration might be (2*R*,3*R*) or (2*S*,3*S*) [22]. According to Gaffield [23], a positive n → π* Cotton effect at high wavelength (*ca*. 300–340 nm) indicates a 2*R*-configuration and, consequently, a negative n → π* Cotton effect hinted at 2*S*. The experimental CD spectrum of **1** showed a positive Cotton effect around 340 nm and a negative one at 296 nm (Figure 3). This, in combination with the above described ^1^H-NMR results permitted assignment of a 2*R*,3*R*-configuration to compound **1**. This assumption was confirmed by the CD spectrum of the structurally related (but monomeric) flavanone (2*R*,3*R*)-taxifolin [24], which was similar to that of compound **1**. Compound **1** was, thus, unambiguously identified as 2*R*,3*R*-chromone-3''-hydroxyflavanone and, hence, is a new natural product, subsequently named preussianone. The combination of a chromone-dihydroflavanol is to our knowledge unique in GBs. Only one flavanone-chromone, compound **6**, has so far been isolated from leaves of *Garcinia dulcis* [25]. The biosynthetic origin of the new biflavonoid is so far unknown. In agreement with considerations of the biosynthesis of the related compound **6** [25] the unsubstituted chromone ring could originate from the elimination of a phenolic substituent from a biflavone precursor related to manniflavone (**3**).

The chemical structures of the other four compounds, **2–5** were determined as exemplified for **2** as followed. Compound **2** was obtained as a white powder, [*α*]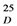 = +3° (*c* = 0.1, MeOH). The ^1^H- and ^13^C-NMR spectra recorded in DMSO-*d*_6_ at room temperature (Table 1 and Table 2) showed two sets of signals (in a 1:1 ratio), while in LC-MS only one single peak was observed. The use of a chiral column under the same separation conditions did not show a splitting, either, which could be related to conformationally semi-stable isomeric structures of compound **2**, possibly atropo-diastereomers. In order to obtain better resolved signals, the NMR spectra were recorded on a 600-MHz spectrometer at 25 °C and at 80 °C. At this high temperature only one set of signals was obtained suggesting that **2** may adopt different conformations at 25 °C, which are in a rapid equilibrium at 80 °C. From the UV maxima and from the ^1^H-NMR, ^13^C-NMR, and HRESIMS data, the base structure of **2** corresponded to 3'',4',4''',5,5'', 5''',7,7''-octahydroxy-biflavanone, which has previously been isolated from *Cratoxylum neriifoliu* [26]*.* In order to assign whether the monomers were attached via a C-3/C-6'' or a C-3/C-8'' linkage, **2** was *O*-methylated and carbon chemical shifts recorded in DMSO-*d_6_* were compared. The permethylated compound displayed eight signals below 57 ppm corresponding to the eight methoxylated aromatic groups. Duddeck *et al.* showed that in *ortho*-disubstituted methoxylated C-3/C-6'' biflavonoids, the methoxyl groups appear at low-field with chemical shifts between 59 and 61 ppm.

**Table 1:** ^1^H-NMR (600 MHz) data of **2** in DMSO-*d*_6_ (*δ*, ppm). Variable temperature at 25 °C and 80 °C. **2a,b** denote the two conformers present at 25 °C.

No.	Compound (Temp.)		
	2a (25 °C)	2b (25 °C)	2 (80 °C)
H-2	5.34	5.68	5.52
H-3	4.66	4.44	4.57
OH-5	12.48	12.20	12.03
H-6	5.88	5.88	5.83
H-8	5.94	5.84	5.91
H-2'	7.11	7.11	7.08
H-3'	6.76	6.64	6.72
H-4'	9.55	9.49	10.24
H-5'	6.76	6.64	6.72
H-6'	7.11	6.64	7.08
H-2''	4.98	4.85	4.92
H-3''	4.18	3.94	4.08
OH-5''	11.75	11.85	11.61
H-6''	5.88	5.88	5.92
H-2'''	6.65	6.63	6.73
H-3'''	6.79	6.58	6.85
H-4'''	8.99	8.99	9.11
H-5'''	8.88	8.88	8.37
H-6''	6.84	6.76	6.73

**Table 2:** ^13^C-NMR data of **2** DMSO-*d*_6_ (*δ*, ppm). **2a,b** are related to the two conformers present at 25 °C.

No.	Compounds (Temp. °C)		
	2a (25 °C)	2b (25 °C)	2 (80 °C)
C-2	81.6	81.3	81.2
C-3	47.2	47.1	47.2
C-4	196.6	196.5	195.7
C-5	163.5	163.8	162.3
C-6	96.0	96	94.5
C-7	166.4	166.3	165.6
C-8	94.8	94.9	95.7
C-9	162.7	162.5	163.5
C-10	101.2	101.1	101.1
C-1'	127.8	127.9	127.7
C-2'	128.9	128.9	128.2
C-3'	115.5	118.9	114.4
C-4'	157.8	157.6	157.2
C-5'	115.5	118.9	114.4
C-6'	128.9	128.9	128.2
C-2''	82.7	82.7	82.8
C-3''	72.3	71.9	71.9
C-4''	197.4	197.4	196.6
C-5''	161.8	162.2	162.4
C-6''	96.0	95.8	95.4
C-7''	162.1	161.7	164.2
C-8''	94.8	94.9	99.78
C-9''	160.1	159.4	161.6
C-10''	100.1	99.5	100.9
C-1'''	128.1	128.2	127.8
C-2'''	118.9	117.3	114.8
C-3'''	115.1	115.0	114.8
C-4'''	145.8	144.5	144.7
C-5'''	144.9	145.3	144.7
C-6'''	115.3	115.0	114.9

In compound **2**, these values were below 57 ppm. In accordance with [27] it was deduced that the two flavanone moieties were attached via C3 and C-8'' [27]. The main HMBC correlations within the flavanone moiety of **2** were similar to those of **1** (Figure 2) and supported the linkage as C-3/C-8''. Based on the comparison of the [*α*]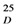 , UV, and ^1^H-NMR data (Table 1) with those reported in the literature [7,28], compound **2** was assigned as (+)-GB2. In a similar way, compounds **3** and **4** were identified as manniflavanone and (−)-GB1, respectively. Compound **5**, named GB2a, with [*α*]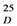 = +6° (*c* = 0.1, MeOH) was found to possess a molecular formula of C_30_H_22_O_11_ (HRESIMS). The ^1^H- and ^13^C-NMR data were similar to those previously reported in the literature by several authors [26].

### 2.2. Absolute Configurations of Compounds *2–5*

The determination of the absolute configurations at C-3 and C-8'' of biflavonoids and the revision of their stereostructures have recently been under discussion [29,30,31,32]. Different configurations were postulated, including controversial ones. Yuanqing *et al*. demonstrated that empirical rules derived from monomeric flavonoids may not be applicable to the configurational assignment of complex 3,8''-biflavonoids [30]. The absolute configurations of (+)-GB2 (**2**) and (−)-GB1 (**4**) have already been revised in the literature as 2*R*,3*S*, 2''*R*,3''*R* [3,29].

For manniflavanone (**3**) and (+)-GB2a (**5**), however, so far no CD data have been recorded. The CD spectra of all these compounds have now revealed Cotton effects corresponding to the electronic n → π* and π → π* transitions of the two moieties.

The CD curve of manniflavanone (**3**) was similar to those of **2** and **4**. According to the rule of Gaffield [22], the positive n → π* transition at 330 nm and the negative π → π* transition at 320 nm, evidenced a (2''*R*,3''*R*)-configuration at the 3-hydroxyflavanone moiety in **2**, **4**, and **3**. The negative n → π* transition at 302 nm and the positive π → π* transition at 292 nm of the 3-substituted flavanone part suggested a common (2*R*,3*S)*-configuration for these three compounds. The CD data of manniflavanone (**3**), (+)-GB2 (**2**), and (−)-GB1 (**4**) were identical, so that the absolute configuration of **3** was assigned as 2*R*,3*S*,2''*R*,3''*R*, which is in agreement with the literature data [3,29].

**Figure 4:**
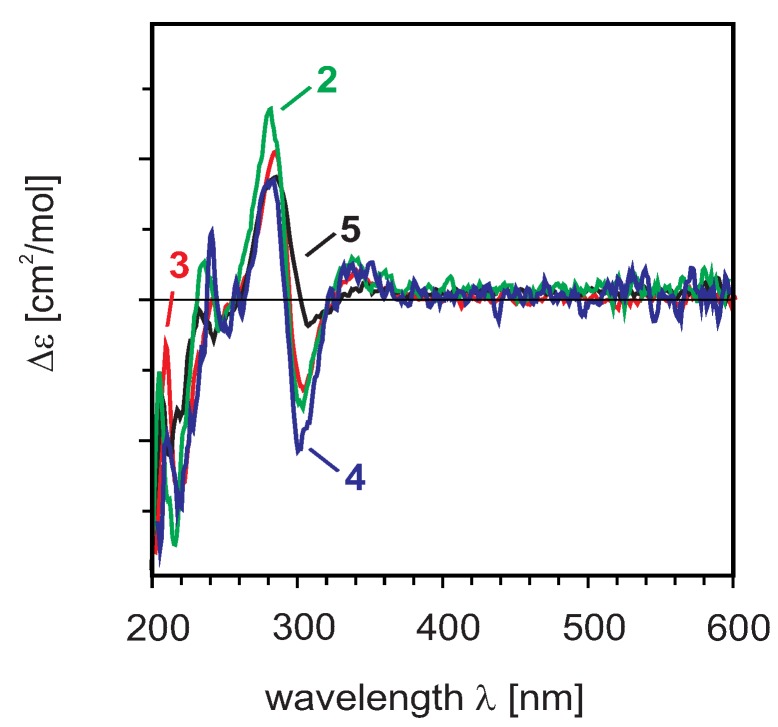
Experimental CD spectra of compounds **2–5**.

The experimental CD spectrum of GB2a (**5**) was identical to that of the related biflavanone *ent*-naringeninyl-(I-3α,II-8)-4'-*O*-methylnaringenin [31]. Following the Gaffield rules, the absolute configuration of GB2a (**5**) was found to be 2*R*,2''*S*,3*S* (Figure 4).

### 2.3. Biological Activity

The antibacterial activities of the extract and of the isolated compounds were examined. The extract of *G. preussii* showed an effect against *E. coli*, *P. aeruginosa*, *S. aureus*,and *E. faecalis*. Among the pure isolated compounds, only **2** and **3** displayed any antibacterial activity (Table 3). Their MIC values were moderate compared to those of amentoflavone and 4'-methoxyamentoflavone isolated from *G. livingstonei*, which exerted excellent antibacterial activity against *E. coli* and *E. faecalis* with MIC values of 6 and 8 μg/mL respectively [11]. The new chromone biflavone did not significantly contribute to the biological activity of the extract.

**Table 3:** Antibacterial activity of *G. preussii* extract (μg/mL).

Compounds	*E. coli*	*P. aeruginosa*	*S. aureus*	*E. faecalis*
**2** (+)-GB2	>512	>512	>512	>512
**3** manniflavanone	>512	>512	256	>512
Extract	>128	>512	Nt	Nt
Gentamycin	1.0	1.0	1.0	16.0

Nt: non tested.

## 3. Experimental

### 3.1. General

1D and 2D NMR spectra (^1^H, ^13^C, COSY, HSQC and HMBC) were recorded at 25 °C on a Varian Unity Inova 500 MHz spectrometer, using DMSO-*d_6_* as solvent. For comparison purposes, NMR experiments were also performed at 25 °C and 80 °C in DMSO-*d_6_* on a Bruker DMX 600 MHz instrument. HRESIMS were determined on a Waters Micromass LCT-Premier time-of-flight mass spectrometer (Milford, MA, USA) equipped with an electrospray interface (ESI). LC/UV-MS analysis was carried out on an HPLC 1100 system equipped with a photodiode array detector (Agilent technologies). For analytical HPLC, a Symmetry^®^ C_18_ column (250 × 4.6 mm i.d., 5 µm, Waters) was used, with H_2_O/MeOH (0.1% FA) as the mobile phase. The following gradient, 30–70 during 30 min, was applied at a flow rate of 0.8 mL/min. The detection was performed at 254, 280, and 360 nm. Under the same separation conditions a Lux Cellulose-1A chiral column from Phenomenex^®^ (4.6 × 250 mm; 5 μm) was used. MPLC was performed using a Büchi 681 pump equipped with a Knauer UV detector using a RP 18 LiChropep (40–60 µm; 460 × 50 mm i.d., Merck). The detection was performed at 254 nm. Column chromatography was carried out using RP18 LiChroprep (15–25 µm, Merck) silica gel 60 (0.063–0.200 mm, Merck), and Diaion HP20/L. UV spectra were recorded on a UV/vis lambda 20 (Perkin Elmer). CD spectra were taken on a J-715 spectropolarimeter at room temperature by using a 0.1 cm standard cell and spectrophotometric-grade MeOH, and are reported in ∆*ε* values (cm^2^/mol) at the given wavelength *λ* (nm).

### 3.2. Plant Material

*Garcinia preussii* (Clusiaceae) leaves were collected in July 2008 at Ngoumé in the Central Province of Cameroon. The identification was done by the Cameroon National Herbarium, where the voucher specimen (55520/HNC) was deposited for future reference.

### 3.3. Extraction and Isolation

Dried leaves of *Garcinia preussii* (5 kg) were extracted at room temperature successively with acetone (3 × 5 L) and methanol (3 × 5 L). The methanol extract (50 g) was submitted to flash chromatography on TLC silica gel 60 H (15 µm) eluted with a CH_2_Cl_2_/methanol mixture of increasing polarity to give four main fractions: F1 (645 mg), F2 (5.09 g), F3 (6.20 g), and F4 (7.60 g). F4 (5 g) was chromatographed on a Sephadex LH-20 column eluted with methanol to yield seven fractions: A (128 mg), B (534 mg), C (85.4 mg), D (38 mg), E (24 mg), F (30 mg), and G (2.5 g). Fraction A–G were filtered on a polyamide cartridge (polyamide 6 d50, 49, 50) with methanol/H_2_O (95:5) to remove the dark coloration and tannins. Fraction G was repeatedly purified by chromatography on a silica gel column with CHCl_3_/methanol (9:1) to give compound **2** (150 mg) and **3** (200 mg).

F3 (5 g) was applied on a Sephadex LH-20 column using methanol as the eluent to give seven additional fractions (I-VII). Fraction VII (2.5 g) was separated on a silica gel open column with an isocratic system (CHCl_3_/MeOH). Compounds **1** (48 mg), **4** (100 mg), and **5** (30 mg) were purified from this last step.

### 3.4. Preussianone

(**1**): Yellow oil. [*α*]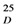 +48.7 (*c* 0.1, MeOH); UV (MeOH) λ_max_ (logε) nm 258 (4.05), 291 (4.00), 326 (3.58), 386 (2.76); IR (crystal) *ν_max_* cm^−1^ 3,080, 2,259, 2,129, 1,616, 1,506, 1,436, 1,363, 1,207, 1,131, 974. Negative-HRESIMS *m/z* 479.0589 [M-H]^−^ (calcd for C_24_H_15_O_11_ 479.0614); CD (MeOH; *c* = 0.003472 mol/L): ∆ε = 208 (−2.18), 236 (1.75), 296 (−3.56) cm^2^/mol. For ^1^H-NMR (DMSO-*d*_6_) and ^13^C-NMR (DMSO-*d_6_*), see Table 4.

**Table 4:** ^1^H- and ^13^C-NMR data of compound **1** (recorded at 500/125 MHz in DMSO-*d*_6_; *δ* 25 °C, ppm; *J*, Hz).

No.	C	H
2	82.9	4.95 (*d*, 1H, 10.7 Hz)
3	71.6	4.43 (*d*, 1H, 10.7 Hz)
4	198.0	-
5-OH	162.8	11.90
6	95.74	6.08 (*s*, 1H)
7	165.1	-
8	98.2	-
9	160.6	-
10	100.4	-
1'	127.9	-
2'	115.0	6.67 (*brs*, 1H)
3'	144.7	-
4'	145.6	-
5'	119.0	6.67 (*brs*, 1H)
6'	115.1	6.80 (*brs*, 1H)
2''	156.5	8.13 (*s*, 1H)
3''	115.1	-
4''	179.8	
5''-OH	161.6	12.70
6''	98.9	6.19 (*d*, 1H, 2.1 Hz)
7''-OH	164.2	-
8''	93.7	6.34 (*d*, 1H, 2.1 Hz)
9''	157.5	-
10''	104.3	-

### 3.5. Antimicrobial Assay

The bacterial reference strains used in this study, *Escherichia coli* (ATCC 25922), *Pseudomonas aeruginosa* (ATCC 27853), *Enterococcus faecalis* (ATCC 29212), and *Staphylococcus aureus* (ATCC 29213), were obtained from the HUG (Geneva University Hospital, Geneva, Switzerland). Mueller-Hinton broth (MHB, Oxoid) and Mueller-Hinton agar (MHA, BioMérieux) were used as liquid and solid medium, respectively. All strains were grown for 24 h at 37 °C. The minimum inhibitory concentration (MIC) of the various compounds were determined by using the broth dilution method in 96-well microtiter plates as previously described [33]. Gentamycin (Sigma-Aldrich) was used as control. The concentration range was between of 0.0625 and 32 μg/mL. The obtained values were in agreement with the reference [34]. The MIC corresponds to the lowest concentration of the compounds that inhibits visible growth (visual turbidity). The growth was detected by the reduction of INT into formazan, a red-purple molecule. The highest dilution of a compound in which no red-purple color appears corresponds to its MIC.

## 4. Conclusions

The new compound preussianone (**1**) is the second representative of a rare type of chromone flavanone to be reported in *Garcinia* genus. Other four known bioflavonoids were also identified in the leaves of *Garcinia preussii*. The availability of a set of related GBs permitted a first comparative CD investigation of these compounds allowing assignment of their absolute configurations as 2*R*,3*S*,2''*R*,3''*R* for **2–4** and 2*R*,3*S*,2''*S* for **5**. The present study thus supports the recent establishment of the absolute configurations of bioflavonoids, but revises the assignment (2*S*,3*R*,*2''R*,*3''R*) previously obtained for GBs [3,35]. The observed antibacterial activity of the extract in the traditional use of the plant may be a consequence of the imaginable synergetic contribution of the ensemble of metabolites.

## References

[1] Williams C.A., Harborne J.B., Dey P.M., Harborne J.B. (1989). Biflavonoids. Methods in Plant Biochemistry.

[2] Kim H., Park H., Son K., Chang H., Kang S. (2008). Biochemical pharmacology of biflavonoids: Implications for anti-inflammatory action. Arch. Pharm. Res..

[3] Iwu M.M., Igboko O.A. (1990). Biflavonoid constituents of *Garcinia kola* roots. Fitoterapia.

[4] Geiger H., Quinn C., Harborne J.B., Mabry T.J., Mabry H. (1975). Biflavonoids. The Flavonoids.

[5] Geiger H., Quinn C., Harborne J.B., Mabry T.J. (1982). Biflavonoids. The Flavonoids.

[6] Ferreira D., Slade D., Marais J.P.J. , Øyvind M.A., Markham K.R. (2006). Bi-, Tri-, Tetra-, Penta-, and Hexaflavonoids. The Flavonoids: Chemistry, Biochemistry and Applications.

[7] Amellal M., Bronner C., Briancon F., Haag M., Anton R., Landry Y. (1985). Inhibition of mast-cell histamine-release by flavonoids and biflavonoids. Planta Med..

[8] Geiger H., Quinn C., Harborne J.B. (1988). Biflavonoids. The Flavonoids.

[9] Braide V.B. (1993). Anti-inflammatory effect of Kolaviron, bio-flavonoid extract of *Garcinia kola*. Fitoterapia.

[10] Cowan M.M. (1999). Plant products as antimicrobial agents. Clin. Microbiol. Rev..

[11] Kaikabo A.A., Eloff J.N. (2011). Antibacterial activity of two biflavonoids from Garcinia livingstonei leaves against Mycobacterium smegmatis. J. Ethnopharmacol..

[12] Louh G.N., Lannang A.M., Mbazoa C.D., Tangmouo J.G., Komguem J., Castilho P., Ngninzeko F.N., Qamar N., Lontsi D., Choudhary M.I. (2008). Polyanxanthone A, B and C, three xanthones from the wood trunk of *Garcinia polyantha* Oliv. Phytochemistry.

[13] Chen J.-J., Ting C.-W., Hwang T.-L., Chen I.-S. (2009). Benzophenone derivatives from the fruits of *Garcinia multiflora* and their anti-inflammatory activity. J. Nat. Prod..

[14] Lin Y.-M., Anderson H., Flavin M.T., Pai Y.-H.S., Mata-Greenwood E., Pengsuparp T., Pezzuto J.M., Schinazi R.F., Hughes S.H., Chen F.-C. (1997). *In vitro* anti-HIV activity of biflavonoids isolated from *Rhus succedanea* and *Garcinia multiflora*. J. Nat. Prod..

[15] Bouquet A. (1969). Féticheurs et Médecines Traditionnelles du Congo (Brazzaville).

[16] Visser L.E. (1975). Plantes Médicinales de la Côte d'Ivoire.

[17] Cotterill P.J., Scheinmann F., Stenhouse I.A.  (1978). Extractives from Gurriferae 34. Kolaflavanone, a new biflavanone from nuts of *Garcinia kola* HECKEL-applications of C-13 nuclear magnetic-resonance in elucidation of structures of flavonoids. J. Chem. Soc. Perkin Trans. 1.

[18] Jackson B., Locksley H.D., Scheinmann F., Wolstenholme W.A. (1971). Extractives from guttiferae. Part XXII. The isolation and structure of four novel biflavanones from the heartwoods of *Garcinia buchananii* Baker and *Garcinia. eugeniifolia* Wall. J. Chem. Soc. C.

[19] Markham K.R., Sheppard C., Geiger H. (1987). ^13^C-NMR studies of some naturally occurring amentoflavone and hinokiflavone biflavonoids. Phytochemistry.

[20] Chari V.M., Ilyas M., Wagner H., Neszmélyi A., Fa-Ching C., Li-Kuang C., Yu-Chin L., Yu-Meei L. (1977). ^13^C-NMR spectroscopy of biflavanoids. Phytochemistry.

[21] Terashima K., Kondo Y., Aqil M., Waziri M., Niwa M. (1999). A study of biflavanones from the stems of *Garcinia kola* (Guttiferae). Heterocycles.

[22] Slade D., Ferreira D., Marais J.P.J. (2005). Circular dichroism, a powerful tool for the assessment of absolute configuration of flavonoids. Phytochemistry.

[23] Gaffield W. (1970). Circular dichroism, optical rotatory dispersion and absolute configuration of flavanones, 3-hydroxyflavanones and their glycosides: Determination of aglycone chirality in flavanone glycosides. Tetrahedron.

[24] Hosoi S., Shimizu E., Ohno K., Yokosawa R., Kuninaga S., Coskun M., Sakushima A.  (2006). Structural studies of zoospore attractants from *Trachelospermum jasminoides* var. *pubescens*: Taxifolin 3-*O*-glycosides. Phytochem. Anal..

[25] Ansari W.H., Rahman W., Barraclough D., Maynard R., Scheinmann F. (1976). Biflavonoids and a flavanone chromone from leaves of *Garcinia dulsis* (Roxb) Kurz. J. Chem. Soc. Perkin Trans. 1.

[26] Kumar V., Brecht V., Frahm A.W. (2004). Conformational analysis of the biflavanoid GB2 and a polyhydroxylated flavanone-chromone of *Cratoxylum neriifolium*. Planta Med..

[27] Duddeck H., Snatzke G., Yemul S.S. (1978). ^13^C-NMR and CD of some 3,8''-biflavanoids from *Garcinia* species and of related flavanone. Phytochemistry.

[28] Crichton E.G., Waterman P.G. (1979). Manniflavanone, a new 3,8-linked flavanone dimer from the stem bark of *Garcinia mannii*. Phytochemistry.

[29] Ferrari J., Terreaux C., Kurtán T., Szikszai-Kiss A., Antus S., Msonthi J.D., Hostettmann K. (2003). Isolation and on-Line LC/CD analysis of 3,8'-linked biflavonoids from *Gnidia involucrata*. Helv. Chim. Acta.

[30] Ding Y., Xing-Cong L., Ferreira D. (2007). Theoretical calculation of electronic circular dichroism of the rotationally restricted 3,8"-biflavonoid morelloflavone. J. Org. Chem..

[31] Mbwambo Z.H., Kapingu M.C., Moshi M.J., Machumi F., Apers S., Cos P., Ferreira D., Marais J.P.J., Vanden Berghe D., Maes L. (2006). Antiparasitic activity of some xanthones and biflavonoids from the root bark of *Garcinia livingstonei*. J. Nat. Prod..

[32] Li X.-C., Joshi A.S., Tan B., ElSohly H.N., Walker L.A., Zjawiony J.K., Ferreira D. (2002). Absolute configuration, conformation, and chiral properties of flavanone-(3 → 8'')-flavone biflavonoids from *Rheedia acuminata*. Tetrahedron.

[33] Wiegand I., Hilpert K., Hancock R.E.W. (2008). Agar and broth dilution methods to determine the minimal inhibitory concentration (MIC) of antimicrobial substances. Nat. Protoc..

[34] Eloff J.N. (1998). A Sensitive and quick microplate method to determine the minimal inhibitory concentration of plant extracts for bacteria. Planta Med..

[35] Sonnenbichler J., Madubunyi I., Scheer H. (1987). Stereochemistry of 2-hydroxybiflavanonols from *Garcinia kola* nuts. Z. Naturforsch..

